# How phagocytic cells kill bacteria: Lessons from a professional killer

**DOI:** 10.1002/2211-5463.70280

**Published:** 2026-06-27

**Authors:** Otmane Lamrabet, Pierre Cosson

**Affiliations:** ^1^ Department of Applied Biology, College of Sciences University of Sharjah University City UAE; ^2^ Faculty of Medicine, Department of Cell Physiology and Metabolism University of Geneva Switzerland

**Keywords:** antibacterial mechanisms, bacterial killing, *Dictyostelium discoideum*, innate immunity, lysosomal enzymes, phagocytosis

## Abstract

Professional phagocytic cells such as neutrophils and macrophages, as well as free‐living soil amoebae like *Dictyostelium discoideum*, employ evolutionarily conserved mechanisms to ingest and kill bacteria. Ingested bacteria are killed in phagosomes by four main antibacterial systems: production of reactive oxygen and nitrogen species, accumulation of bactericidal ions, deployment of lysosomal enzymes, and release of membrane‐permeabilizing peptides. Despite extensive studies, important questions persist. How complete is the list of known antibacterial mechanisms? What is their relative contribution to bacterial destruction? How specific are they for different bacterial species? This review examines how *D. discoideum* amoebae and mammalian phagocytes ingest and eliminate non‐pathogenic bacteria, focusing on three specific elements: the dual role of lysozyme, the specificity of antibacterial mechanisms, and the redundancy of the antibacterial arsenal. Overall, our current knowledge highlights the sophisticated strategies employed by phagocytes against diverse microorganisms. It also provides a framework to study the more complex interactions between phagocytic cells and pathogenic bacteria.

## Graphical abstract

How phagocytic cells ingest and kill bacteria has been studied for more than a century, but many questions remain unanswered. The study of the amoeba *Dictyostelium discoideum* brings new answers, and new questions.

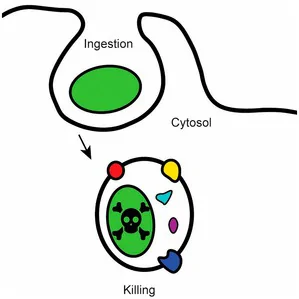

Phagocytosis is the phenomenon by which eukaryotic cells ingest big particles, typically with a diameter of at least 1 μm, such as microorganisms or cell debris. In the human body, it is common to distinguish professional phagocytic cells, whose main function is to ingest and kill infectious microorganisms, from non‐professional phagocytes, which can internalize particles under specific conditions but are not specialized for this task. Professional phagocytes include mostly circulating neutrophils and macrophages. The human body produces 100 billion short‐lived circulating neutrophils each day, and most of them die within a day without ever ingesting a microorganism [1, 2]. Tissue‐resident macrophages or monocyte‐derived macrophages, by contrast, have much longer lifespans (several months or years) and are more likely to encounter at least a few microorganisms over their lifespan. Non‐professional phagocytic cells include a wide range of cell types, including but not limited to many epithelial cells or fibroblasts. Beyond multicellular organisms, the environment is also teeming with free‐living phagocytic cells, in particular amoebae. These cells feed upon microorganisms which they ingest, kill, and digest efficiently [3]. The most studied amoeba is *Dictyostelium discoideum*. In laboratory conditions, a *D. discoideum* amoeba can ingest up to one bacterium every minute over its whole exponential growth. It amply deserves to be called a professional killer. To the best of our knowledge, *D. discoideum* amoebae and mammalian phagocytes appear to mobilize very similar mechanisms to ensure chemotaxis, phagocytosis, and intracellular destruction of bacteria [4, 5].

Ingestion of large particles by phagocytic cells requires a massive change in cell shape, accomplished by actively reorganizing the actin cytoskeleton [6]. Following internalization, the formed phagosome rapidly fuses with lysosomes and evolves into a mature phagolysosome where ingested microorganisms are killed, destroyed, and digested [7]. The mechanisms allowing mammalian phagocytic cells to kill ingested bacteria have been described in numerous excellent reviews on the subject [4, 5, 8, 9, 10, 11]. These reviews provide a description of the main killing mechanisms that have been identified and studied over more than a century, which can be grouped in four main categories: (i) production of reactive oxygen and nitrogen species; (ii) transport and accumulation of bactericidal ions, including protons, within the phagolysosome; (iii) digestive lysosomal enzymes; and (iv) membrane‐permeabilizing proteins or peptides. Although a large number of antibacterial mechanisms have been identified, each one is complex and often incompletely understood. Additionally, many other general questions remain unanswered. For example, how close to completion is the already extensive list of antibacterial mechanisms? What is the relative importance and contribution of each killing mechanism? Are different bacteria killed by different mechanisms?

In this review, we describe fundamental lessons drawn from the study of the amoeba *D. discoideum*, and what it taught us on the function of phagocytic cells. First, we describe the surprisingly complex role of lysozyme. Second, we address the question of the specificity of killing mechanisms for distinct bacterial targets. Third, we consider how redundant the antibacterial arsenal is. Finally, we discuss how research on *D. discoideum* could advance our understanding of bacterial pathogenicity and host–pathogen interactions.

## Disentangling the two functions of lysozyme

**Fig. 1:**
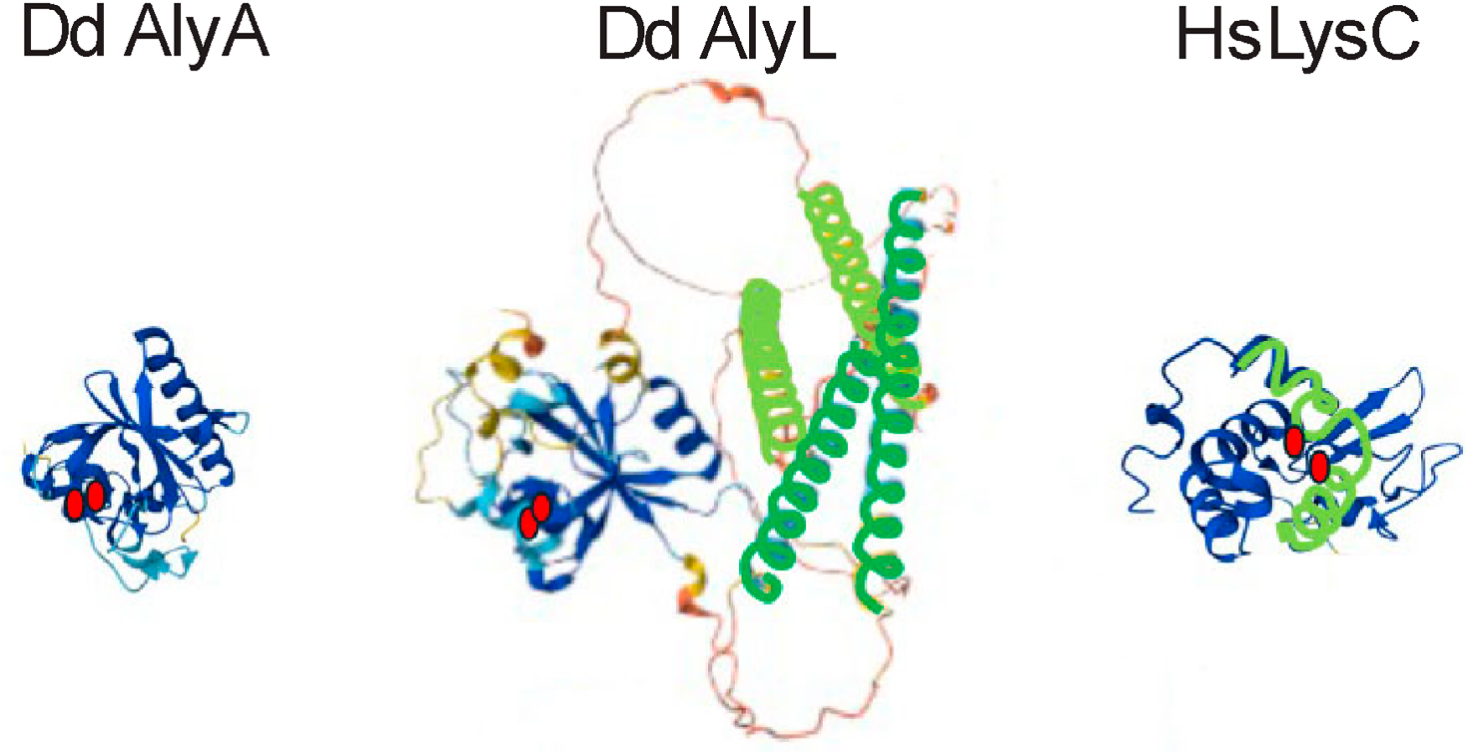
Structure of human and amoeba lysozymes. The structure of the *D. discoideum* (Dd) AlyA and AlyL amoeba lysozymes and human lysozyme (Hs LysC) as predicted by AlphaFold [15] is shown. The position of the two key residues of the active enzymatic site is indicated (red ovals). In human LysC and *D. discoideum* AlyL lysozymes, the putative membrane‐permeabilization domain is highlighted in green. The structures have been simplified for clarity.

Lysozyme was first described in 1922 as a bacteriolytic substance present in many human body fluids such as nasal mucus, tears, or saliva and capable of lysing the Gram‐positive bacteria *Micrococcus lysodeikticus* [12]. It was then identified in many vertebrates and can notably easily be isolated from the hen egg where it represents more than 3% of the total protein content [13]. Humans have only one classical (C‐type) bacteriolytic lysozyme (LysC); mice have two (LysM and LysP). Both species also exhibit several other lysozyme‐like proteins with specialized functions [14]. In different species, lysozymes can exhibit very divergent sequences and structures, and this was used to define six main families of lysozymes (chicken C‐, goose G‐, viral V‐, invertebrate I‐, entamoeba‐, and Aly‐type). The only conserved feature in all families is the presence of two key residues in the catalytic center, one glutamic acid and one aspartic acid (Fig. 1). Lysozyme was later shown to hydrolyze peptidoglycans, a key structural component of bacterial cell walls [16, 17]. In addition, its role in immune defense was demonstrated by several studies *in vivo*. In mice, genetic inactivation of Lysozyme M significantly reduced the lung clearance of infecting Gram‐positive *Streptococcus pneumoniae* bacteria and increased their lethality [18]. On the contrary, overexpression of lysozyme in mouse lungs protected them from streptococcal infections [19]. On the basis of these cumulative observations, it was reasonable to assume that lysozyme kills Gram‐positive bacteria, such as *M. lysodeikticus* and *S. pneumoniae*, by digesting their outer protective layer of peptidoglycan.

However, two key findings challenged this model. First, lysozyme also kills Gram‐negative bacteria efficiently both *in vitro* [20] and *in vivo* [21], which was somewhat surprising, since in Gram‐negative bacteria, the layer of proteoglycans is located in the intermembrane space and thus not accessible to extracellular lysozyme. Second, an enzymatically inactive mutant of mouse lysozyme retains its bacteriolytic activity against Gram‐positive and Gram‐negative bacteria both *in vitro* and *in vivo* [22]. Together, these observations indicated that the bacteriolytic activity of vertebrate lysozyme is not due, at least not solely, to its enzymatic activity but also to its ability to permeabilize membranes. Although the structural basis of this activity remains incompletely defined, it supports a dual function model in which enzymatic and membrane‐disruptive activities coexist (Fig. 1). The relative contribution of these two mechanisms may depend on the bacterial envelope: in Gram‐positive bacteria, where the peptidoglycan layer is thick and directly accessible, enzymatic hydrolysis is likely a major determinant of bacteriolysis, whereas in Gram‐negative bacteria, the outer membrane limits access to peptidoglycan and membrane‐permeabilizing activity becomes more important. Alternatively, the bacteriolytic activity of lysozyme may be due exclusively to its non‐enzymatic membrane‐permeabilizing properties. More detailed and quantitative studies may be necessary to clarify this point.

Due to the ubiquitous presence of peptidoglycans in bacterial cell walls, lysozymes are also frequently observed in organisms that feed on bacteria, such as amoebae. The *D. discoideum* genome encodes at least 22 putative lysozymes belonging to four different families (chicken C‐, entamoeba‐, viral V‐, and Aly‐type) [17, 23]. This unusually large repertoire likely reflects the need for functional diversification to cope with the broad diversity of bacterial prey encountered in the soil environment. The study of lysozyme in the *D. discoideum* amoeba is much more recent than in mammals: a seminal study identified AlyA as the main proteoglycan‐degrading enzyme in *D. discoideum* [24] and showed that it belonged to a new family of lysozymes, called the Aly (Amoeba‐lysozyme) family. The *D. discoideum* genome encodes eight Aly proteins (A, B, C, D1, D2, E, F, and L) [17], two of which (AlyA and AlyL) have been studied in detail. Genetic inactivation of AlyA caused a significant decrease in cellular proteoglycan‐degrading activity (> 60%) [24, 25] while loss of AlyL had no measurable effect on total enzymatic activity [23, 25]. Remarkably, killing of ingested *K. pneumoniae* bacteria in phagosomes was significantly slower in *alyL* KO cells than in WT cells, but unaffected in *alyA* KO cells [25]. This indicates that, like in vertebrates, amoeba lysozymes have two distinct functions: degradation of proteoglycans (main function of AlyA) and destruction of bacteria (main function of AlyL). The existence of lysozymes specialized in one or the other function provided clues as to their mode of action: AlyA and AlyL both contain an Aly‐type enzymatic domain, and AlyL exhibits, in addition, a separate domain containing four large amphipathic helices (Fig. 1). Complementation experiments confirmed that the four amphipathic helices, and not the enzymatic activity, are responsible for the bacteriolytic activity of AlyL [25]. Phylogenetic analyses indicate that the bacteriolytic module of AlyL was secondarily acquired and appended to an ancestral Aly enzymatic core [17, 25]. This modular organization demonstrates that peptidoglycan hydrolysis and bactericidal activity are structurally and functionally separable processes in *D. discoideum*. The recent biochemical characterization of two *D. discoideum* Entamoeba‐type lysozymes (CF45‐1 and CF50) further illustrates the diversity of lysozyme functions: these two proteins exhibit lysozyme enzymatic activity in different physicochemical conditions, with CF50 showing particularly strong activity at acidic pH. To date, the bactericidal role (if any) of CF45‐1 and CF50 remains untested [26]. Additional experiments will be necessary to determine if the many lysozymes in *D. discoideum* constitute a highly diverse and adaptable antibacterial system, or if some lysozymes serve other functions.

Collectively, studies in vertebrates and *D. discoideum* converge on a striking conclusion: lysozyme possesses two mechanistically distinct functions, peptidoglycan hydrolysis and bacterial membrane permeabilization. Remarkably, despite this functional convergence, the structural organization of the vertebrate and amoeba lysozymes differ profoundly (Fig. 1). The folding of the vertebrate and amoeba enzymatic domains is entirely different. The bacteriolytic element is a putative helix at the surface of the vertebrate lysozyme and an extended set of helices forming a separate domain in the *D. discoideum* AlyL (Fig. 1). This situation is a remarkable example of convergent evolution in which both animal lysozymes and a *D. discoideum* lysozyme combine a lysozyme enzymatic domain with a membrane‐permeabilizing region. The recurrent association of these two functions strongly suggests a selective advantage. Peptidoglycan degradation may facilitate access of the permeabilizing module to the membrane of Gram‐positive bacteria. In Gram‐negative bacteria, permeabilization of the outer membrane may enable the enzymatic lysozyme domain to reach otherwise shielded peptidoglycan. This structural integration could represent an evolutionary adaptation allowing lysozyme to attack a variety of diverse bacterial cell walls. Further investigation into the precise mechanisms by which the two domains of lysozyme interact functionally and contribute to bacterial killing could provide insights into the evolutionary pressures shaping bacteriolytic mechanisms in phagocytic cells. The unsuspected complexity of the mode of action of lysozyme may be more common than we think: nothing in the structure of the lysozyme indicated *a priori* its ability to lyse membranes independently from its enzymatic activity. *D. discoideum* amoebae may be a good model to test further this hypothesis, since at least the AlyL bacteriolytic domain is actually quite evident in the protein structure (Fig. 1). One possibility would be to identify and study other phagolysosomal enzymes exhibiting similar domains, and to evaluate their potential to participate in bacterial killing and their mode of action. This strategy may allow us to reveal if the dual mode of action of lysozyme is the rule or an exception.

## Intracellular killing: Specificity and redundancy

The capacity of phagocytic cells to recognize and eliminate a wide variety of microorganisms is a hallmark of innate immunity. Ingested microorganisms are presumably exposed in phagosomes to a similar range of bacteriolytic mechanisms. However, increasing evidence indicates that bacterial killing is not uniform and that different molecular mechanisms destroy different bacteria. This killing specificity has been partially observed in mammalian phagocytes and is increasingly being explored in unicellular models such as amoebae.

While the list of mammalian bactericidal effectors is long and diverse, it remains unclear how specific each one is for a subset of bacteria. Scattered evidence suggests that specificity is the rule rather than the exception. For example, *in vitro*, different bacteria survive very differently when exposed to different concentrations of hydrogen peroxide (H_2_O_2_): in one study, 0.1% H_2_O_2_ was sufficient to kill *E. coli* bacteria, while 2–3% was necessary to kill *Listeria monocytogenes* or *Staphylococcus aureus* [27]. Similarly, genetic inactivation of *nox2* in mice, encoding superoxide‐producing NADPH oxidase, makes neutrophils incapable of producing superoxide, and this renders patients susceptible to infections with only a subset of bacteria, notably *S. aureus* [28]. These observations suggest strongly that reactive oxygen species are more important for the killing of certain microorganisms both *in vitro* and *in vivo*. However, it is difficult to unify these observations into a complete description, and today, we do not have a clear evaluation of how important reactive oxygen species are for the elimination of different bacterial species in phagocytic cells.

In mammalian phagocytes, the multilayered antibacterial arsenal is also believed to exhibit functional redundancy, as evidenced by extensive genetic and pharmacological studies. Inhibiting or genetically ablating a single antimicrobial effector, such as NADPH oxidase (as in chronic granulomatous disease), myeloperoxidase, specific proteases (e.g., neutrophil elastase), or lysozyme, does not abolish microbicidal activity entirely [29, 30]. As discussed below, this observation suggests that the killing of a given bacteria is not achieved by a single killing effector (e.g., lysozyme or reactive oxygen species), but rather by several independent mechanisms.

An ideal experiment would be to generate a set of mutant phagocytic cells, deleting one specific effector in each of them and to determine how efficiently different types of bacteria are killed in each mutant cell. This is difficult to achieve with mammalian phagocytes, in particular because fully differentiated phagocytic cells (neutrophils and macrophages) do not divide, and because many mutations may alter the differentiation of phagocytic cells. On the contrary, *D. discoideum* provides an ideal system to generate specific mutant cells and to analyze quantitatively their ability to kill different bacteria. When this systematic analysis was conducted in *D. discoideum*, several mutants were defective for killing of *K. pneumoniae* and *E. coli* [25]. AlyL was the most important effector of *K. pneumoniae* killing, but it was dispensable for the killing of the other bacteria, indicating that AlyL is specifically involved in the killing of *K. pneumoniae* bacteria. BpiC, a lipopolysaccharide (LPS)‐binding protein, was necessary for efficient killing of both *K. pneumoniae* and *E. coli* but not *B. subtilis. In vitro* experiments also suggested that different effectors participate in the lysis of different bacteria: bacteriolytic activity against different bacteria was found in different purified fractions of *D. discoideum* extracts [31]. Together, these observations demonstrate that bacterial killing in *D. discoideum* relies on a combinatorial set of effectors, each contributing to the killing of a subset of bacteria.

**Fig. 2:**
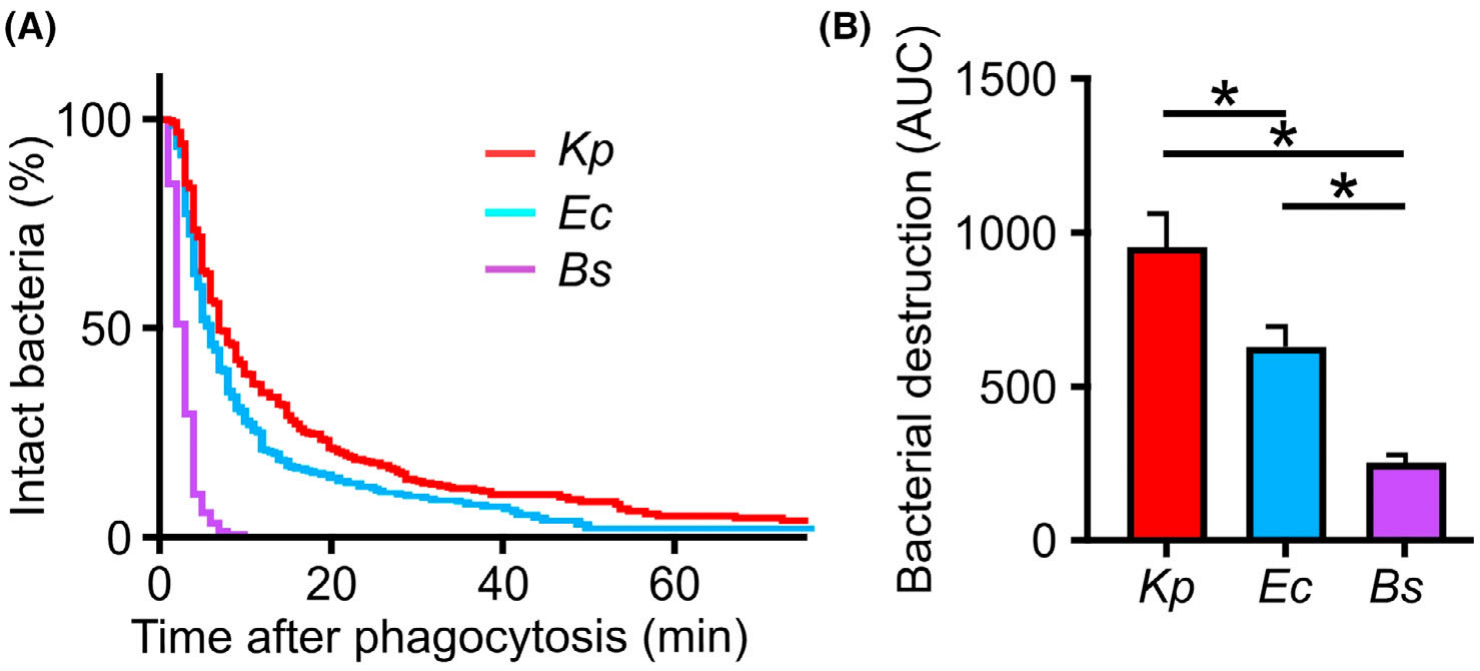
Kinetics of intracellular killing in *D. discoideum* phagosomes. (A). The kinetics of intracellular destruction of *K. pneumoniae* (Kp), *E. coli* (Ec), and *B. subtilis* (Bs) were measured in parallel as described previously [25]. (B). The killing of different bacteria was quantified in multiple experiments, by measuring the area under the survival curve (AUC) for each bacteria. *B. subtilis* was significantly faster than that of *K. pneumoniae* and *E. coli* (mean ± SEM. *: *P* < 0.05, Mann–Whitney test, Kp: *N* = 11; Ec: *N* = 7; Bs: *N* = 8).

Remarkably, as seen in mammalian phagocytic cells, all *D. discoideum* mutant cells analyzed retained the ability to kill bacteria, albeit less efficiently [25, 31, 32, 33, 34]. This suggests that no single antibacterial factor is essential for killing, and that bacterial destruction is achieved by the cumulative effect of many antibacterial effectors, which can function independently of each other. Observations of the killing of *Bacillus subtilis* bacteria suggest a high degree of functional redundancy: not a single *D. discoideum* mutant showed any defect in the killing of this bacteria. The first interpretation of this result is that there is an as yet unidentified mechanism specifically responsible for the destruction of *B. subtilis*. The second interpretation is that *B. subtilis* bacteria are killed so efficiently in phagosomes that no single effector is essential for rapid killing. This second interpretation is supported by the observation that *B. subtilis* is killed faster than other bacteria in phagosomes (Fig. 2).

In summary, in both amoebae and mammalian phagocytes, the built‐in redundancy in killing mechanisms ensures robustness of innate immunity. Even when a pathogen evolves resistance to one specific pathway (e.g., toxic reactive oxygen species like superoxide), other killing mechanisms can compensate and maintain effective microbial killing. It should, however, be stressed that the observations of bacterial killing by phagocytic cells are still very fragmentary, limited to the study of a few bacteria and a few bactericidal mechanisms. Further experiments will be necessary both in amoebae and in human phagocytic cells to extend these early observations. It will be particularly interesting to establish the degree of specificity and redundancy of different effectors at play in the killing of different species of bacteria. Understanding how non‐pathogenic bacteria are killed will also be essential to understand how pathogenic bacteria resist killing, as discussed in the next section.

## Pathogenic bacteria

The situations described above refer mostly to confrontations between a non‐pathogenic bacterium and a phagocytic cell equipped to kill it efficiently. In such cases, the phagocytic cell is expected to eliminate rapidly ingested bacteria. In the human body, this elimination prevents the development of an infection, whereas in the environment, it allows amoebae to feed and proliferate. The situation becomes more complex when pathogenic bacteria are considered, and this has become a very active field of investigation.

In human patients, pathogenic bacteria are, by definition, capable of causing harmful infections. It is, however, difficult to deduce the virulence of a bacterium from its infectious history in a patient. The pathogenicity of a bacterium is thus often tested by infecting in a controlled manner animals, particularly mice. By definition, a pathogenic bacterium harms or kills an infected mouse and a non‐pathogenic bacterium does not. It is experimentally demanding and ethically problematic to test virulence of a bacterium in mice. These difficulties have led to the use of alternative host models to measure the pathogenic potential of bacteria. This strategy is based on the (probably oversimplistic) notion that pathogenic bacteria use the same mechanisms to infect a human patient, a drosophila fly, or an amoeba. Amoebae have been used as a means of testing the pathogenic potential of a wide array of pathogenic microorganisms. In this model organism, innocuous bacteria can be used as food by amoebae, and bacteria capable of perturbing host physiology are expected to perturb its feeding process. According to this definition, pathogenic bacteria were defined as bacteria inhibiting the growth of amoebae [35, 36, 37]. As a general rule, the pathogenicity of a bacterium against amoebae correlates well with its pathogenic potential in mammals [35, 36, 37]. It is very simple to measure virulence of a bacterium against *D. discoideum* amoebae and hundreds of strains can be tested in parallel. In this model system, large‐scale testing allowed to isolate bacterial mutants with altered virulence or chemical compounds altering bacterial virulence [38, 39, 40, 41]. Cumulated evidence indicates that amoebae can be used as a simple model organism to study bacterial virulence, confirming the notion that bacterial virulence against different hosts relies largely on the same virulence mechanisms. This is clearly a simplification, and there may be another interesting facet to this discussion, as developed below.

Importantly, many pathogenic bacteria, often designated as opportunistic pathogens, live largely or exclusively in the environment and only exceptionally infect human patients. These include several of the pathogens prioritized in the WHO BPPL 2024 update such as *K. pneumoniae*, *Acinetobacter baumannii*, and *Pseudomonas aeruginosa* [42]. In the environment, these bacteria are engaged in a classical predator–prey relationship with amoebae, creating conditions conducive to Darwinian selection: only bacteria capable of evading capture and killing by phagocytic amoebae can thrive in the environment. The concept of coincidental evolution proposes that the same mechanisms used to escape phagocytic amoebae also confer to the bacteria the ability to escape the human innate immunity and to invade the human body [37]. In addition, opportunistic pathogens must also adapt to different selection conditions as they transition from the environment to the human body. This presumably explains why some pathogenic bacterial features like LPS‐associated O‐antigens [43, 44], capsule [45], or quorum sensing [46, 47] can be downregulated or even lost during infection of a patient, due to accumulation of mutations in the bacterial genome. These virulence factors are probably useful for bacterial survival in the amoeba‐infested environment. In the human body, they may be less advantageous, energetically costly, or even detrimental by increasing immune recognition. Some bacteria such as *Legionella pneumophila* reach a further level of pathogenicity: they adapt to survive and replicate in amoebae, and many of them can also replicate within human macrophages [48, 49, 50].

In summary, pathogenicity should not always be considered only as a trait directed toward human hosts. In many opportunistic pathogens, the emergence of pathogenic traits is rather the result of a strong selection pressure to resist predation by environmental phagocytes such as amoebae. The similarity between human phagocytes and soil amoebae accounts for the fact that bacteria resistant to amoebae are also resistant to innate immunity and capable of mounting infections. What comes out in the flesh is actually sometimes bred in the soil.

## Conflicts of interest

The authors declare no conflict of interest.

## Author contributions

OL and PC jointly conceived the study, conducted the literature review, analyzed and interpreted the data, drafted the manuscript, prepared figures and tables, and critically revised the text. Both authors approved the final manuscript.

## Data Availability

All results discussed in this manuscript were already published elsewhere in cited articles. Only data from Fig. 2 were not published previously, and the full data set is available upon request.
